# Disruption of Cerebellar–Cerebral Functional Connectivity in Temporal Lobe Epilepsy and the Connection to Language and Cognitive Functions

**DOI:** 10.3389/fnins.2022.871128

**Published:** 2022-06-28

**Authors:** Linlin Pang, Binglin Fan, Zirong Chen, Zexiang Chen, Caitiao Lv, Jinou Zheng

**Affiliations:** Department of Neurology, The First Affiliated Hospital of Guangxi Medical University, Nanning, China

**Keywords:** temporal lobe epilepsy, graph theory, language impairment, cognitive impairment, cerebellum, resting-state functional magnetic resonance imaging (rs-fMRI)

## Abstract

**Objective:**

To investigate the changes in the cerebellar-cerebral language network in temporal lobe epilepsy (TLE) patients from the cerebellar perspective, the research analyzes the changes of language and cognitive network in terms of functional connectivity (FC), as well as their efficiency of the reorganization were evaluated basing on relationship between the network metrics and neuropsychological scale scores.

**Methods:**

30 TLE patients and 30 healthy controls were recruited. Brain activity was evaluated by voxel-mirrored homotopic connectivity analysis (VMHC). Two groups were analyzed and compared in terms of language FC using the following methods: Seed-to-Voxel analysis, pairwise correlations [region of interest(ROI)-to-ROI] and graph theory. Correlation analysis was performed between network properties and neuropsychological score.

**Results:**

Compared with healthy participants, VMHC values in the Cerebellum Anterior Lobe, Frontal Lobe, Frontal_Sup_R/L, Cingulum_Ant_R/L, and Cingulum_Mid_R/L were decreased in TLE patients. Decreased FC was observed from the Cerebelum_10_R to the left inferior frontal gyrus, from the Cerebelum_6_R to the left Lingual Gyrus, from the Cerebelum_4_5_R to left Lingual Gyrus, left Cuneal Cortex and Precuneous Cortex, from the Cerebelum_3_R to Brain-Stem, and from the Cerebelum_Crus1_L to Cerebelum_6_R in TLE patients. The FC was enhanced between bilateral Cingulum_Mid and angular gyrus and frontoparietal insular cranium, between Frontal_Sup_Med L and left/right superior temporal gyrus (pSTG l/r), while it was decreased between left middle temporal gyrus and pSTG l/r. Compared with controls, the Betweenness Centrality (BC) of the right superior marginal gyrus (SMG), Temporal_Pole_Mid_R and Temporal_Mid_L as well as the Degree Centrality (DC) and Nodal Efficiency (NE) of the right SMG were lower in TLE patients. Further analysis showed that decreased VMHC in bilateral Cerebellum Anterior Lobe was positively correlated with the Boston Naming Test score in TLE patients, but it was negatively correlated with the Verbal Fluency Test score. The NE and DC of SMG_R were both negatively correlated with visual perception score in Montreal Cognitive Assessment.

**Conclusion:**

Our results suggest that presence of abnormalities in the static functional connectivity and the language and cognitive network of TLE patients. Cerebellum potentially represents an intervention target for delaying or improving language and cognitive deficits in patients with TLE.

## Introduction

Epilepsy is a chronic disease that affects cognitive functions,includinglanguage (Pereira et al., 2010). Patients with temporal lobe epilepsy (TLE) typically suffer from auditory naming problems, visual naming problems, and speech fluency declines; about 33% of these patients have difficulty finding words in spontaneous speech and 40% have dysfunction in naming tasks (Bell et al., 2001, 2003; Lomlomdjian et al., 2011). A previous work (Trimmel et al., 2021) found dyslexia and naming disorder to be a common disabling problem in TLE patients; It demonstrated functional and structural language network impairment. Language processing is predominantly left-lateralized, however individuals with epilepsy showed a larger chance of bilateral or right brain (atypical) linguistic dominance than healthy controls (Goldmann and Golby, 2005). Especially, based on Wada tests and fMRI, up to 33% of right-handed epilepsy patients have been shown to have an atypical verbal advantage (Janecek et al., 2013).

Controlling epileptic seizures in the cerebral cortex and deep brain tissue, has been well investigated. Nevertheless, stimulating brain regions other than the epileptic foci also holds promise (Soper et al., 2016; Elder et al., 2019). One area of interest is the cerebellum. Although the cerebellum is not a region that is traditionally associated with epilepsy (Miterko et al., 2019),and a large number of studies have recently shown that there are close fibrous connections between the cerebellum and a wide range of cortical and subcortical regions through the cerebellum-thalamus-cortex circuit. Combined with changes in the cerebellar activity during epileptic seizures, Martha and colleagues proposed that the cerebellum may be a potential therapeutic target for epileptic control (Blumenfeld et al., 2009; Kros et al., 2017; Streng and Krook-Magnuson, 2021). Beyond its influence on the motor function, the cerebellum expresses an important part of the cognitive processing network (Strick et al., 2009). Different regions of the cerebellar cortex are involved in language-related functions, including language learning, verbal working memory, semantic processing as well as word retrieval and generation (Schmahmann et al., 2019). The specific distribution of motor and non-motor areas in the cerebellar cortex has been determined. In consistence with this topography, language activation was found to be broader and stronger in the right cerebellum, which supports the right lateralization of the cerebellar language function (Guell et al., 2018a). Cerebellar non-motor representations were found to be mainly located in lobule VI, Crus I/II, VIIB, IX, and X, while the regions involved in language processing were shown to correspond to the default mode network (lobule VI, Crus I/II, lobule IX and lobule X) (Buckner et al., 2011; Guell et al., 2018b).

The cerebellum is part of the language network, which is activated in various reading and language tasks. During sentence completion, the right lobule VI, Crus I/II of the cerebellum were shown to be activated in concert with the supratentorial network, which is involved in reading and language processing, including the bilateral middle temporal gyrus (MTG) and inferior frontal gyrus (IFG), and a strong functional connectivity was demonstrated with important nodes in these networks, including the left IFG (Price, 2012). The work of D’Mello et al. demonstrated that transcranial direct current stimulation increased the functional connectivity between the right Crus I and cuneus, between the left inferior frontal gyrus and superior marginal gyrus, and between the left superior marginal gyrus and the left precuneus and right inferior frontal gyrus. Increased connectivity in these regions may facilitate the formation of sentence semantic processing (D’MELLO et al., 2017). Furthermore, the models of lateralized (or maybe bilateral) cerebral representation of language in dextrals and sinistrals seem to be reflected at the cerebellar level by a “lateralized linguistic cerebellum,” subserved by crossed cerebello-cerebral connections between the cortical language network and the cerebellum (Marien et al., 2014). Naming function and language fluency are fundamental to language function, and many studies have reported naming defects in TLE patients. The Boston Naming Test (BNT) have been used to assess language skills and semantic memory by naming images. Language fluency tests were used to assess verbal and semantic memory (Romann et al., 2012). In our previous studies, we have shown a disruption of the cerebellar functional network in patients with right temporal lobe epilepsy (Zhou et al., 2019). However, it remains unclear whether the connection between the cerebellum and the language network is disrupted and how do the changes in the cerebellum network affect the language function in TLE patients.

To approach the above-mentioned questions, in this study, we evaluated rs-fMRI data on language network reorganization in the functional connectivity (FC) of TLE patients. We tried to determine the effects of a dysfunctional cerebellum lateralization on the language networks in TLE patients. In order to obtain the core regions that constitute language components, the language regions of the cerebellum were labeled according to the cerebellum discrete task activity map and resting state map (Buckner et al., 2011; Guell et al., 2018b). Bilateral cerebellar functional regions were selected as regions of interest (ROI) (Marcian et al., 2018). We used the following analyses to assess the cerebellar-cerebral language network in TLE patients: (a) Seed-to-Voxel analysis; (b) ROI-to-ROI analysis; (c) Graph theoretical analysis, which includes efficiency parameters performed at the level of the network and nodes (such that nodes are language network regions), to estimate the possible topological changes that occur on the specific network attributes. We have generated our language and cognitive network, based on the results of FC studies, and the 27 regions of interest within the language network at individual level. Then, Spearman’s correlation was calculated between selected FC parameters and language scale scores to evaluate the effectiveness of FC recombination.

## Materials and Methods

### Participants

Thirty TLE patients with unilateral TLE [including sixteen left-side TLE (LTLE) and fourteen right-side TLE (RTLE)] were recruited in the epilepsy clinic of the First Affiliated Hospital of Guangxi Medical University in the period from September 2019 to September 2020. The TLE patients were diagnosed using the International League Against Epilepsy’s standards (ILAE), which included medical history and seizure semiology, ictal or interictal scalp electroencephalogram (EEG), and magnetic resonance imaging (MRI) (Berg et al., 2010; Scheffer et al., 2017). The inclusion criteria included the following: (1) Ictal or interictal EEG revealed epileptic discharges beginning from a unilateral temporal lobe; (2) the typical clinical semiology was consistent with seizures of temporal lobe origin; (3) regular use of anti-epileptic drugs (AEDs); (4) there were no aberrant structural imaging findings in the brain MRI besides hippocampal sclerosis. The exclusion criteria were as follows: (1) other kinds of systemic, neurological, or psychiatric illnesses; (2) diagnosis of multifocal or extratemporal epilepsy; (3) developmental defects, cortical malformations, or other focal lesions on clinical MRI; (4) severe mental disorder or dementia; (5) lack of cooperation with the inspection; (6) any history of drug or alcohol misuse. This study included thirty healthy adult participants who were matched for age, gender, and education. The research approach was approved by the Ethics Committee at the First Affiliated Hospital of Guangxi Medical University.

### Neuropsychological Examination

Each participant underwent a set of neuropsychological tests, Cognitive abilities, such as language, memory, attention, visual ability and executive function, were assessed using the Montreal Cognitive Assessment (MoCA). According to the Boston Naming Test (BNT), 30 items were selected from an original list of 60 items based on the cultural relevance in China. Starting with the first item, if the participant correctly named the item, the next item was tested. Points were given for self-correction, and if the participant gave an incorrect answer or did not give an answer within 20 s, then semantic cues were given. If the subject could not correctly say the name of the object after being provided with a semantic cue, then no points were scored (Cheung et al., 2004). In the verbal fluency test (VFT), the controlled spoken word association test, the category “fruit” in particular, was used to assess the semantic fluency, which requires the subject to name as many different fruit names as possible in 1 min (Ardila, 2020).

### MRI Data Acquisition

Magnetic resonance imaging data were obtained using the 3.0-T scanner (Philips, Netherlands). The resting-state functional magnetic resonance imaging (rs-fMRI) scanning prolonged for 450 s, and 225 brain volumes were retrieved. During the scanning, participants were reminded to close their eyes, stay awake and not think about anything. Headphones and cushions were utilized for noise mitigation and limitation of head movement. We collected the high-resolution T1-weighted images in sagittal orientation. Then, we used an axial T2 fluid-attenuated inversion recovery sequence to remove the clinically silent brain lesions. The rs-fMRI were collected using the following parameters: repetition time/echo time (TR/TE) = 2,000/30 ms, voxel size = 3.44 mm × 3.44 mm × 4 mm, field of view = 220 mm × 220 mm, matrix size = 64 × 64, flip angle = 90°, number of slices = 41, slice thickness = 3.5 mm, slice gap = 0.5 mm. The used parameters of T1-weighted structural images were as follows: TR/TE = 7.8/3.4 ms, flip angle = 9°, field of view = 256 mm × 256 mm, image matrix = 256 × 256, number of slices = 176 slices, slice thickness = 1 mm, voxel size = 1.0 mm × 1.0 mm × 1.0 mm.

### MRI Data Preprocessing

The Data Processing & Analysis for Brain Imaging (DPABI) software^1^ was used on the Matrix Laboratory (MATLAB) R2018b platform to extract and preprocess the rs-fMRI data. First, we converted the images from the DICOM to NIfTI format. Then, the rest of the images were preprocessed as follows: (1) The first 10 volumes of the remaining data were discarded to ensure magnetization equilibration; (2) The slice time was corrected; (3) The data were checked and corrected for head motion. If the subject exhibited head motion greater than 3.0 mm or head rotations that exceeded 3°, then the data were excluded; (4) Using the new segment and DARTEL technique, the white matter, gray matter, and cerebrospinal fluid from each T1 image were segmented, and then the images were spatially normalized to Montreal Neurological Institute (MNI) coordinates, and the resolution was resampled at 3 mm × 3 mm × 3 mm; (5) The data were spatially smoothed with a 4 mm full width at half- maximum (FWHM) Gaussian kernel; (6) Linear and quadratic trends were excluded; (7) Using linear regression, the covariates, including head motion, white matter, and cerebrospinal fluid signal, were eliminated to minimize interference; (8) The temporal filtering frequency band was limited to a band-pass (0.01–0.10 Hz). There was no head motion more than 3 mm translation and/or three degree rotation in any of the patients. We estimated the mean frame-wise displacement (FD) for each subject and compared them between two groups using a non-parametric test to reduce connectivity measurement artifacts caused by head motion. The TLE group with HCs had no significant difference in mean FD (Table 1).

**TABLE 1:** Demographics, clinical and neuropsychological parameters data of subjects in two groups.

Variables	TLE (*n* = 30)	HC (*n* = 30)	*P*
**Demographic characteristics**			
Age (years)	31.03 ± 9.02	27.27 ± 5.73	0.059*^a^*
Sex (male/female)	12/18	10/20	0.789*^b^*
Education (years) (range)	12(6–16)	12(6–17)	0.970*^c^*
**Clinical features**			
Age at onset (years)	20.06 ± 10.66	NA	NA
Epilepsy duration (years)	10.39 ± 7.58	NA	NA
Seizure frequency (time/months)	3.34 ± 3.82	NA	NA
Seizure type (Focal/FBTCS)	17/13	NA	NA
AEDs (mono-/polytherapy)	12/18	NA	NA
Mean FD (mean ± SD)	0.06 ± 0.04	0.05 ± 0.02	0.408^a^
**Neuropsychological test**			
BNT (range)	24.5(16–30)	29(22–30)	0.000^c^
VFT (range)	10(4–16)	16(8–22)	0.000^c^
MoCA total score	25.73 ± 3.81	28.90 ± 1.49	0.000^a^
Executive function (range)	1(0–1)	1(0–1)	0.073^c^
Fluency (range)	1(0–2)	2(1–2)	0.000^c^
Orientation (range)	6(4–6)	6(5–6)	0.160^c^
Calculation (range)	3(0–3)	3(2–3)	0.083^c^
Abstraction (range)	3(0–3)	3(2–3)	0.006^c^
Delayed recall (range)	3.5(1–5)	4.5(2–5)	0.001^c^
Visual perception (range)	3(1–3)	3(2–3)	0.023^c^
Naming (range)	4(2–4)	4(3–4)	0.126^c^
Attention (range)	2(0–2)	2(0–2)	0.313^c^

*FBTCS, focal to bilateral tonic–clonic seizures; FD, frame-wise displacement; AEDs, antiepileptic drugs; NA, not available; BNT, Boston Naming Test; VFT, verbal fluency test; MoCA, Montreal Cognitive Assessment.*

*^a^P was calculated using the Independent t test.*

*^b^P was calculated using the χ2 test.*

*^c^P was calculated using the Mann–Whitney test.*

### VMHC Analysis

The voxel-mirrored homotopic connectivity (VMHC) analysis was performed using the DPABI software. First, the functional homotopy of each pair of mirrored voxels was calculated using Pearson’s correlation coefficient. The coefficient values were converted to VMHC values using the Fisher z-transformation. In the group comparisons, age, gender, and educational level were included as annoyance covariates. The differences in VMHC between the TLE patient group and the healthy control group were then evaluated using a two-sample *t*-test. We employed a permutation test using Threshold-Free Cluster Enhancement (TFCE), a stringent multiple comparison correction approach, to achieve the best balance between family-wise error rate (sub 5%) and test–retest reliability/replicability due to the limited sample size of our study (Chen et al., 2018). For multiple comparisons, the TFCE with permutation tests were performed at two-tailed *P* < 0.05. The number of permutations were set at 5,000.

### FC Analysis

Before running the FC analyses, we primarily determined the regions of interest (ROIs). The VMHC value of bilateral cerebellum that were related to the scores of BNT and VFT was clearly aberrant. We hypothesized that the bilateral anterior cerebellum or other parts of the cerebellum might be linked to language network, and we decided to investigate further. The CONN toolbox (Whitfield-Gabrieli and Nieto-Castanon, 2012) provides Presupposed 116 ROIs, which make up cortical and subcortical areas from the automated anatomical labeling (AAL) atlas, as well as cerebellar areas from the AAL. Hence, A total of 26 cerebellar areas, were selected as the ROIs. For FC analysis, the CONN toolbox was employed. The seed-based technique was chosen. The data were preprocessed and analyzed using the functional connectivity toolbox “CONN19c”^2^ running on MATLAB R2018b and the Statistical Parametric Mapping method (SPM12^3^). The indices, abbreviations and MNI coordinates of the 26 cerebellar regions used in this study are shown in Supplementary Table 1. Slice-dependent time shifts, systematic odd correction, head spatial registration, spatial smoothing, normalization, and coregistration with structural 3D T1-Weighted were all phases in the resting-state preprocessing. Gray matter, white matter, and cerebrospinal fluid are then separated from the images. We constructed the steps of noise source reduction, first level individual analysis (including correlation analysis) and second level random effect group analysis. Network identification based on seed-based ROIs correlation analysis of the functional connectivity focused on the sensorimotor network, language network, visual network, salience network, and default mode network. After choosing a ROI as the seed, whole-brain correlations time courses can be generated, resulting in spatial maps of the network of interest. To messure functional connectivity, the voxel-wise ROI to ROI correlation maps across the whole brain was calculated. We used linear regression and band pass filtering to remove undesired head movements, physiological effects and other noises. The Fisher R-to-Z transform was used to generate z-plots for each subject using the general linear model weighted regression/correlation measurement of the conditional specific correlation between the ROI time series and time series of each voxel in the whole brain. Finally, the factors of age, sex and education were used as covariables to analyze FC differences between the two groups (*P* < 0.05, FDR corrected).

### Network Construction and Analysis

When CONN was used for FC analysis, the 27 abnormal connections were used to construct a 27 × 27 temporal correlation matrix of the brain. The network is composed of 27 brain regions, including seven brain regions that came from aberrant seed-to-voxel results (Table 3) and 20 brain regions that came from aberrant roi-to-roi results (Figure 4). Most of the 27 brain regions have been reported by two task-fMRI studies, one cross-sectional study for language (Labache et al., 2019) and one analysis for language-and-memory network (Roger et al., 2020). The indices, abbreviations, and MNI coordinates of the 27 abnormal nodes used in this study are shown in Supplementary Table 2.

We used the GRETNA toolbox to construct a functional brain network composed of nodes and edges, with the sparsity ranging from 25 to 50% and a connection density interval of 0.01 (Welton et al., 2020). Each node in the network represented a brain region, while each edge delegated a connection between two nodes. The Pearson correlation coefficient of time series connecting two brain regions was taken as FC and defined as edge, 27 × 27 FC connection matrix was constructed and Fisher’s-Z transformation was performed. Negative functional connections were then set to 0 and only positive functional connections were kept, resulting in an undirected weighted positive resting state functional connection matrix. In order to describe the topology of TLE patients, we calculated the characteristics of the topological network based on the constructed brain network, including small-world, global/local efficiency, degree centrality, intermediate centrality and node efficiency. This helped to determine any significant differences between the groups in the topographic parameters (including SmallWorld, NodalShortestPath, NodalLocalEfficiency, NodalEfficiency, NodalClustCoeff, NetworkEfficiency, DegreeCentrality and BetweennessCentrality). In these network parameters, BC can be defined as the ratio of the total number of the shortest paths in a network to the total number of the shortest paths that pass through this node, reflecting the function and influence of the nodes in the whole network. NE reflects the transmission capacity of local information, while DC expressed as the number of nodes connected to this node. We used GRETNA to calculate the topological property, and then calculated the area under the curve (AUC) of each topological property. Next, we compared AUC values using the two-sample *T* test with the factors of age, sex and education as covariates. We used false detection rate (FDR) correction, and *p* < 0.05 was considered statistically significant. Finally, the results of functional networks were shown using the BrainNet Viewer.

### Statistical Analysis

Demographic, clinical, and neuropsychological data were analyzed using SPSS 23.0 (SPSS Inc., Chicago, IL, United States). Independent sample *T* test was used to analyze the differences between the two groups for data with normal distribution and homogeneity of variance. For data without normal distribution or variance, the Mann–Whitney test was used. The Chi-square test was used for qualitative variable, and pearson’s correlation analysis was used to determine the relationship between the VMHC value, difference node index and language and cognitive function. Statistical significance was set to 0.05.

## Results

### Demographic, Clinical and Neuropsychological Data

Table 1 shows the demographic data and clinical characteristics of 30 TLE patients and 30 HC. There was no statistically significant differences in the age, sex and education level.Based on the neuropsychological tests, we observed that the MoCA total score, BNT and VFT scores of patients with TLE were significantly lower than those of healthy controls.

### VMHC Analysis and Correlation Analysis

**FIGURE 1:**
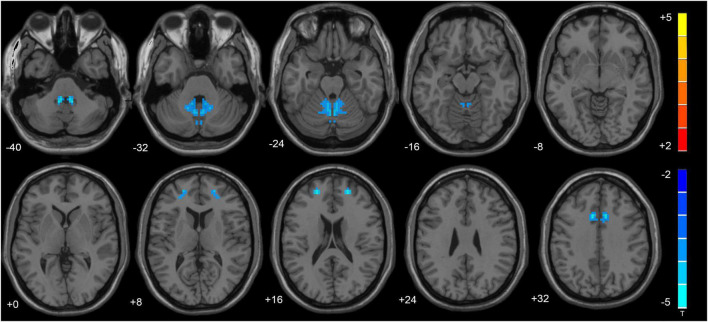
The altered cerebellum and brain regions were revealed in the VMHC analysis. Cold tones shows decreased VMHC.

**FIGURE 2:**
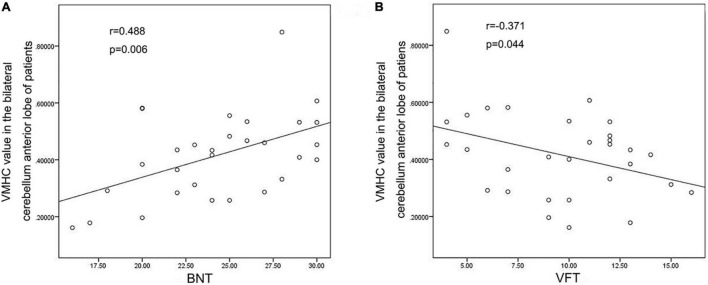
Correlation analysis Results. **(A)** VMHC values of bilateral cerebellum anterior lobe were positively correlated with BNT scores; **(B)** VMHC values of bilateral cerebellum anterior lobe were negatively correlated with VFT scores.

VMHC data analysis showed that the VMHC values in the cerebellum Anterior Lobe, Frontal Lobe, Frontal_Sup_R/L, Cingulum_Ant_R/L and Cingulum_Mid_R/L were decreased in TLE patients compared with healthy controls. Figure 1 and Table 2 show the location and details of the abnormal brain areas. Correlation analysis explored the correlation between the neuropsychological test scores and VMHC abnormal brain region. VMHC value of the bilateral Cerebellum Anterior Lobe were positively correlated with BNT scores of TLE patients (*R* = 0.488, *P* = 0.006, Figure 2). But VMHC value of the bilateral Cerebellum Anterior Lobe were negatively correlated with VFT scores of TLE patients (*R* = −0.371, *P* = 0.044, Figure 2). However, no correlation was observed between the VMHC values of abnormal brain region and MoCA scores.

**TABLE 2:** Brain regions showing significant differences in VMHC between the TLE and HC groups.

Brain regions (AAL)	MNI coordinates			Cluster size
	
	X	Y	Z	
Cerebellum Anterior Lobe	± 6	−42	−39	121
Frontal Lobe	± 18	33	15	24
Frontal_Sup_R/L	± 21	51	18	27
Cingulum_Ant_R/L	± 9	30	18	32
Cingulum_Mid_R/L	± 9	18	33	41

*AAL, automated anatomical labeling; MNI, Montreal Neurological; permutation test 5,000 次, p<0.05, and cluster-level: p < 0.05.*

**TABLE 3:** Seed regions with reduced functional connectivity in TLE vs. HC.

Seed region	Cluster coordinates	Cluster size	Cluster regions	Cluster *p*-Value (FDR)
Cerebelum_10_R	−54 + 16 + 24	98	Inferior Frontal Gyrus Left	0.035
Cerebelum_6_R	−8 −82 −20	93	Lingual Gyrus Left	0.046
Cerebelum_4_5_R	−6 −70 −6	90	Lingual Gyrus Left Cuneal Cortex Left Precuneous Cortex	0.049
Cerebelum_3_R	+ 2 −22 −18	89	Brain-Stem	0.041
Cerebelum_Crus1_L	+ 22 −60 −20	108	Cerebellum 6 Right	0.028

### FC Analysis Results

#### Seed-to-Voxel Results

**FIGURE 3:**
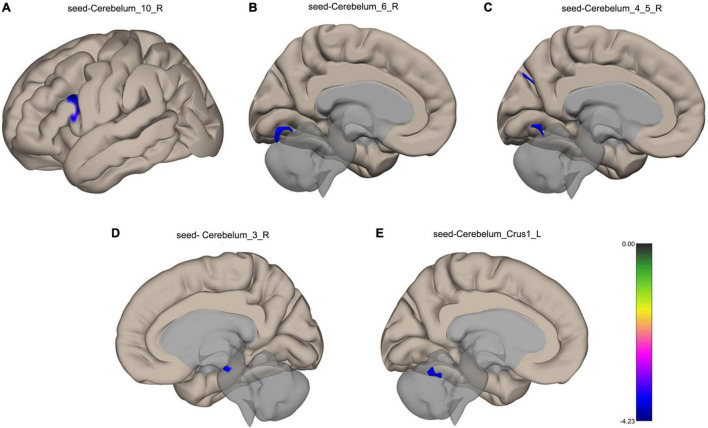
Surface maps for the between-group comparisons of functional connectivity. **(A)** Decreased functional connectivity from the Cerebelum_10_R to the left Inferior Frontal Gyrus in TLE patients. **(B)** Decreased functional connectivity from the Cerebelum_6_R to the left Lingual Gyrus in TLE patients. **(C)** Decreased functional connectivity from the Cerebelum_4_5_R to the left Lingual Gyrus, left Cuneal Cortex and Precuneous Cortex in TLE patients. **(D)** Decreased functional connectivity from the Cerebelum_3_R to the Brain-Stem in TLE patients. **(E)** Decreased functional connectivity from the Cerebelum_Crus1_L to the Cerebellum 6 R in TLE patients. The blue color shows a decreased functional connectivity. FDR correction, *p* < 0.05.

VMHC analysis showed significant abnormalities in bilateral cerebellar areas. The following anomalies were observed: Compared with the healthy participants, TLE patients showed decreased functional connectivity from the Cerebelum_10_R to the left Inferior Frontal Gyrus, from the Cerebelum_6_R to the left Lingual Gyrus, from the Cerebelum_4_5_R to the left Lingual Gyrus, left Cuneal Cortex and Precuneous Cortex, from the Cerebelum_3_R to the Brain-Stem in TLE patients, and from the Cerebelum_Crus1_L to the Cerebellum 6 R, as shown in Figure 3. Table 3 lists the specific details for the brain regions that exhibited abnormal FC strength.

**FIGURE 4:**
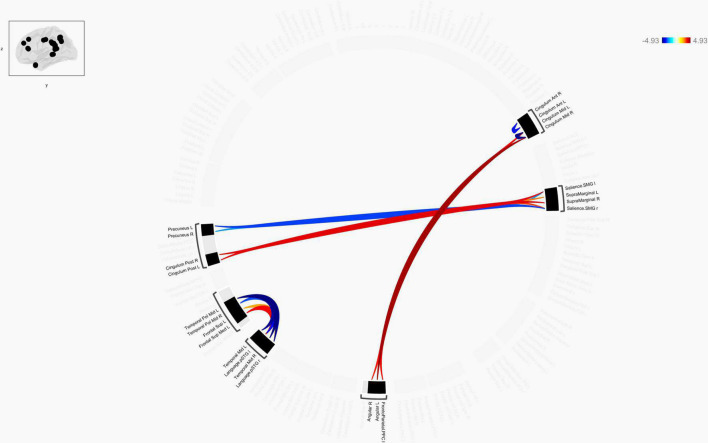
Chord diagram of the ROI-to-ROI FC z-score showing significant group difference between TLE patients and HCs. The abbreviation outside the circle stand for the AAL cerebrum regions. The red line stands for increased FC and the blue line stands for decreased FC in TLE patients compared to HCs. Abbreviations: ROI, region-of-interest; FC, functional connectivity; TLE, temporal lobe epilepsy; HCs: Healthy Controls.

#### ROI-to-ROI Results

Connection diagrams between TLE patients (*n* = 30) and healthy controls (*n* = 30) showed significant differences in the functional connectivity. Specifically, it showed a string diagram of the ROI-to-ROI analysis after FDR correction of the *p-*value. The functional connectivity between bilateral Cingulum_Mid and bilateral angular gyrus and frontoparietal insular cranium was enhanced in TLE patients compared with the control group, while the functional connectivity between the left temporal pol middle gyrus and left/right superior temporal gyrus (Language.pSTG l/r) was decreased. Furthermore, functional connectivity between Frontal_Sup_Med L and left/right superior temporal gyrus (Language.pSTG l/r) was enhanced, as shown in Figure 4. The language network, composing with bilateral inferior frontal gyrus (IFG) and bilateral superior temporal gyrus, were selected from the default automated template available in the CONN toolbox.

### Nodal Properties of Functional Brain Networks

**FIGURE 5:**
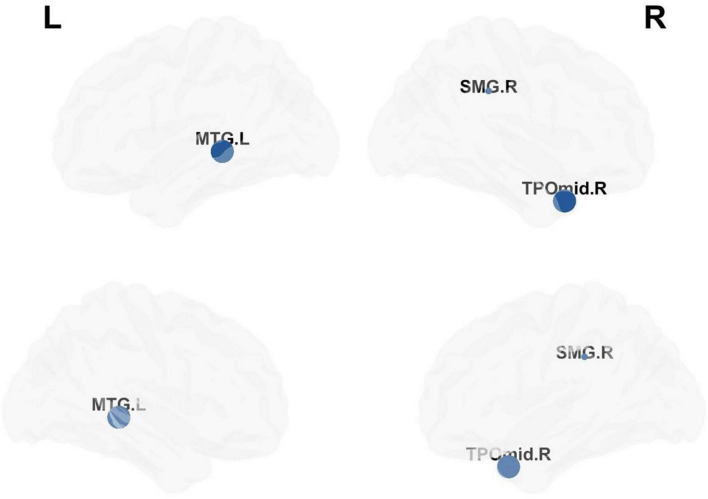
Analysis of nodal properties. The regional distribution with differences in the Betweenness Centrality, Degree Centrality and Nodal Efficiency drawn by the BrainNet Viewer toolbox.

Compared with matched networks, both TLE patients and HCs exhibited the characteristics of a small world network, and no statistically differences were observed in the global properties between the patients and HCs. In order to investigate the alterations in nodal strength in TLE patients, we analyzed several nodal characteristics. The Betweenness Centrality (BC) of the right superior marginal gyrus (SMG), Temporal_Pole_Mid_R (TPOmid.R) and Temporal_Mid_L (MTG.L) were significantly lower in TLE patients compared with controls. The Degree Centrality (DC) and Nodal Efficiency (NE) of the right SMG were significantly lower in TLE patients than in controls (*p* < 0.05 following FDR correction; Figure 5 and Table 4).

**TABLE 4:** Attribute changes of the nodes.

Nodal properties	Node	*TLE*	*HCs*	*P*	*P* (FDR correction)
Betweenness Centrality	SupraMarginal_R	1.547 ± 1.147	2.675 ± 1.975	0.009	0.013^+^
	Temporal_Pole_Mid_R	1.230 ± 1.398	2.202 ± 2.214	0.047	0.047^+^
	Temporal_Mid_L	2.687 ± 1.874	4.517 ± 2.496	0.002	0.007^+^
Degree Centrality	SupraMarginal_R	2.092 ± 0.542	2.450 ± 0.633	0.022	0.022
nodal efficiency	SupraMarginal_R	0.154 ± 0.014	0.163 ± 0.016	0.026	0.026

*+, represented heterogeneity of variance or non-normal distribution of data, permutation test (number of 5,000) P < 0.05.*

### Correlation Between the Network Metrics and Neuropsychological Scale Scores

**FIGURE 6:**
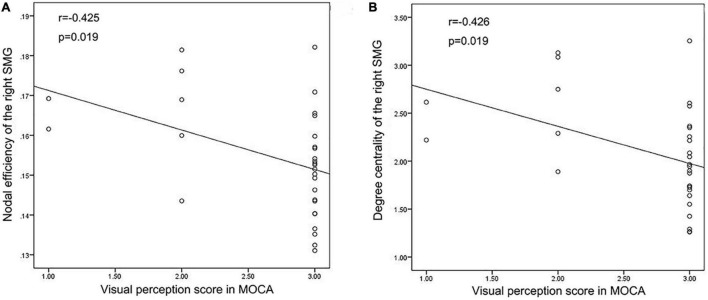
Correlation analysis results. **(A,B)** Correlation analysis showed that Nodal efficiency and Degree centrality of the right SMG were negatively correlated with the visual perception score in MoCA in TLE patients.

We performed Pearson’s correlation analysis between the nodal network parameters with significant differences and MoCA score, BNT score and VFT score. The results showed a negative correlation between the NE of the right SMG and the visual perception score in MoCA as well as between DC of the right SMG and the visual perception score in MoCA in TLE patients. There was no correlation between the network metrics and language scale scores. The results are shown in Figure 6.

## Discussion

From a cerebellar standpoint, the study’s first key goal was to evaluate the changes in the cerebellar-cerebral language network in patients with temporal lobe epilepsy (TLE). Overall, our data point to a pattern of hubs disruption across a local network. In comparison to the control group, TLE patients’ language network hubs have a topological property disturbance (see Figures 3–5). Furthermore, aberrant network measurements had a strong relationship with neuropsychological scale scores.

The hippocampus is typically thought to be the center of problems in TLE, but the variety of tasks related with cerebellar activity is impressive, including language, attention, executive function, and working memory tests (Streng and Krook-Magnuson, 2021). In this study, we specifically found the VMHC values of bilateral anterior cerebellar lobe and superior frontal gyrus decreasing in TLE patients. The cerebellum is supposed to influence cognitive skills, including language ability, according to Petra’s research. During a verbal fluency challenge, they discovered that the left fronto-parietal cortex and the right cerebellum were active, proving that the cerebellum is involved in language skills (Hubrich-Ungureanu et al., 2002). By cross-anatomic associations with the left frontal, parietal, and temporal lobes that are associated with language dominance, multiple meta-analyses confirmed the activation of the right cerebellum in language tasks, with the right cerebellum VI and CrusI involved in language functions and the left VI involved in visuospatial processing (Stoodley and Schmahmann, 2009; E et al., 2014). In addition, studies on patients with speech apraxia after left insula infarction revealed a strong association between the left frontal lobe, where language dominates, and the right cerebellum. Besides, SPECT scans after the disease showed a hypoperfusion in the left inferior frontal and anterior central gyrus and the right cerebellum (Marien et al., 2001). Neuroimaging findings (Guell et al., 2018a) support right-lateralization of language function in the cerebellum, and our study also demonstrated that seeds found in the right cerebellum is wider. Overall, these studies suggest that bilateral cerebellum, especially the right cerebellum, is involved in the disruption of language networks. In line with our results, we observed different patterns of FC changes according to the cerebellum lateralization. Right cerebellum exhibited more pronounced cerebellar–cerebral FC reorganization in comparison with left cerebellum. The higher the VMHC value, the better the coordination of functional connections between hemispheres. In recent studies by our group, we suggested that VMHC abnormalities on behalf of an important fMRI feature, and the VMHC analysis confirmed the changes in VMHC and FC between specific brain regions in TLE patients (Shi et al., 2021). In this study, the decrease in VMHC values in bilateral cerebellum and bilateral superior frontal gyrus suggested an abnormal functional homotopy regulation. The VMHC value of the bilateral Cerebellum Anterior Lobe were positively correlated with BNT score and negatively correlated with the VFT score of TLE patients. Therefore, the abnormal internal functional coordination of bilateral anterior cerebellar lobe and Cingulum_Ant may be one of the reasons behind the impairment of the naming function in TLE patients.

The inferior frontal gyrus delegates an important part of the prefrontal cortex, and its posterior part is involved in language functions. The bilateral superior temporal and inferior frontal cortex represent the neural basis of information processing, which is in turn the cornerstone of language functions. The semantic processing and syntactic processing ability of the top-down mode were mainly achieved by the left inferior frontal cortex, such that the second stage of language network development is closely related to the functional characteristics and structural connections of the left inferior frontal brain region (Skeide and Friederici, 2016). In language processing and social cognition, the cerebellum does not only interact with the prefrontal lobe, but also with the temporal lobe, especially with the CrusI and CrusII lobules (Sokolov et al., 2012, 2014). The study of Booth et al. (2007) used dynamic causal model analysis to show that there are interactive connections between the cerebellum and the left inferior frontal gyrus and left lateral temporal cortex, which are considered to be related to voice processing (Booth et al., 2007). The medial occipital lobe, which is composed of the lingual gyrus and cuneus, is also functionally relevant to the tasks of visual memory storage, visual image, creative thinking and language processing. The fibrous connections between the lingual gyrus and cuneus and the superior temporal gyrus and inferior frontal gyrus support their importance in non-visual functions, including language and memory (Palejwala et al., 2021). A previous meta-analysis reported that the cuneiform and lingual gyrus showed consistent activation in processing language tasks, especially the left lingual gyrus and left cuneus (Zhang et al., 2019). Furthermore, the activity of the different functional modules that are required to perform language processes is assumed to be synchronized and coordinated by the cerebellum (Marien et al., 2014). Hence, the cerebellum acts as a monitor of the execution sequence of language processes, integrating its activities with the so-called “frontal system.” In fact, all cognitive theories strongly rely on the cross-functional linkages of the cerebellum through which it mediates supratentorial cognitive functions (Miterko et al., 2019). Therefore, we hypothesize that the naming capability and language fluency defects in TLE patients may be related to the functional disconnection between the cerebellum and left inferior frontal gyrus, left lingual gyrus and left cuneus.

In TLE, our ROI-to-ROI analysis revealed enhanced functional connectivity between bilateral Cingulum Mid and bilateral angular gyrus and frontoparietal insular cranium regardless of epilepsy lateralization. Roger et al. (2020) did a study that supports this phenomena. ROI-to-ROI and GT analyses in their study also revealed improved language-and-memory network-FC inside limbic regions in mTLE, but these findings were unrelated to epilepsy lateralization. The facilitation of epileptic activity propagation in the brain could be due to left asymmetry, which would explain the broader changes in the left hemisphere (LH) (Ridley et al., 2015). The right hemisphere (RH) would play a protective role, preventing mTLE seizures from spreading to other cortices and compensating for seizures-induced brain dysfunctions (Besson et al., 2014). The two concepts, left hemisphere stimulation of seizure spreading and right hemisphere seizure protection, may be complimentary rather than competing. LH anterior temporal lobe is crucial for semantic processing in reading for individuals with left-sided language processing, while the RH anterior temporal lobe processes semantic information as well. When the anterior temporal lobe in either the left or right brain is damaged, semantic and language processing is predicted to rely more on the contralateral cerebral hemispheres. The equipotentiality model of linguistic proposes that if the linguistic dominant hemisphere is damaged early in development, then another hemisphere will develop language processing ability insteadly (De Bode et al., 2015). The negative impacts of epileptic activity in the temporal lobe may cause restructuring of function from one hemisphere to another, according to these studies (Neudorf et al., 2020).

In our study, the results of functional connectivity analysis suggest a possible existence of compensatory restructuring mechanisms. However, Englot et al. (2016) proposed that the role of the FC increase in dysfunctional areas may be related to the generation of epileptic seizures contagion, rather than acting as a compensatory mechanism during epileptic seizures. Previous histology studies have shown that, while epileptic episodes cause neuron death, they are replaced by new excitable synapse and axonal germination, a mechanism called “reactive plasticity.” This reactive plasticity phenomena helps to scientifically explain by the increase in FC found in dysfunctional regions (Ben-Ari et al., 2008).

In this study, we investigated the topological alterations of functional brain networks in TLE patients using graph theory. All the above-mentioned indicators can effectively reflect the importance of a single node in the network. Previous studies showed that the topological properties of the language and memory network are disrupted in TLE patients. However, no statistically significant differences were found in the naming and verbal fluency performance between TLE patients and healthy controls according to language and cognitive scale scores. Meanwhile, the parameter of NE was negatively correlated with cognitive scores (Roger et al., 2020). Our results are consistent with the above-mentioned findings, as we found that the regions with significantly reduced DC and NE were located in the right SMG, TPOmid.R and MTG.L. As part of the dorsal lexical pathway, SMG plays an important role in converting written language components into phonetic phonemes (Price, 2012). The main function of SMG is proposed to be voice representation before voice generation (Binder, 2017). Young and colleagues (Woo et al., 2016) found that SMG may cause speech impairment related to BP1-2 CNVs. In addition, Roberta et al. (2020) proposed that parietal lobe regions, including the SMG, angular gyrus and precuneus, are involved in the functions of language, visual-spatial cognition and attention orientation. Our results are consistent with previous studies, suggesting that the right SMG and bilateral middle temporal gyrus play a key role in language and cognitive networks in TLE patients.

There are has several limitations to this study. Firstly, the number of participants was limited. More participants should be included in future research to investigate the effects of unilateral temporal lobe epilepsy on brain networks. Secondly, the lateralization of TLE patients was not considered in the subgroup analysis. The course of the disease, seizure frequencies and drug regimens were different among the patients recruited in our research. These clinical factors may lead to changes in the intrinsic functional activity, and further studies should be designed to conduct subgroup analyses accordingly. Finally, longitudinal observation and increasing the sample size should be achieved to explore the damage and compensation mechanisms of language defects, and to establish reliability and stability.

## Conclusion

In conclusion, this study could help our understanding of topological changes of the brain connectivity in TLE patients. The study of resting-state FC through the different cerebellar–cerebral pathways reveals abnormal connectivity changes in patients in regions and hubs traditionally involved in language and cognitive functions. To the best of our knowledge, this is the first study to examine alterations in the FC of cerebellar–cerebral language networks in patients with TLE from a cerebellar perspective. We found decreased functional connectivity between the right cerebellum and left frontal, parietal and temporal lobes. We also observed a correlation between neuropsychological tests and the destruction of the functional network topological property. Furthermore, increased FC between the bilateral Cingulum_Mid and bilateral angular gyrus and frontoparietal insular cranium may exert a compensatory effect on language and cognitive deficits in patients with this pathological condition. In a nutshell, these findings contribute to a better understanding of the disrupted and compensatory features of cerebellar–cerebral network, as well as language and cognitive deficiencies in TLE, and they suggest that the cerebellum could be a target for delaying or improving these clinical deficits in TLE patients.

## Data Availability Statement

The raw data supporting the conclusions of this article will be made available by the authors, without undue reservation. The original contributions presented in the study are included in the article/Supplementary Material.

## Ethics Statement

The studies involving human participants were reviewed and approved by Ethics Committee at the First Affiliated Hospital of Guangxi Medical University. The patients/participants provided their written informed consent to participate in this study. Written informed consent was obtained from the individual(s) for the publication of any potentially identifiable images or data included in this article.

## Author Contributions

LP: writing–original draft and experimental design. BF: experimental design. ZRC: data curation. ZXC: investigation. CL: methodology. JZ: funding acquisition. All authors finally agreed to publish this manuscript.

## Conflict of Interest

The authors declare that the research was conducted in the absence of any commercial or financial relationships that could be construed as a potential conflict of interest.

## Publisher’s Note

All claims expressed in this article are solely those of the authors and do not necessarily represent those of their affiliated organizations, or those of the publisher, the editors and the reviewers. Any product that may be evaluated in this article, or claim that may be made by its manufacturer, is not guaranteed or endorsed by the publisher.

## Footnotes

1
http://rfmri.org/dpabi

2
http://www.nitrc.org/projects/conn

3
http://www.fil.ion.ucl.ac.uk/spm
