# Supplementary material for: Disruption of Cerebellar–Cerebral Functional Connectivity in Temporal Lobe Epilepsy and the Connection to Language and Cognitive Functions
Source: Front Neurosci. 2022 Jun 28;16:871128. doi: 10.3389/fnins.2022.871128 (PMC9273908; doi:10.3389/fnins.2022.871128)
Supplement: Supplementary file 1 [file Table_1.DOCX]

Table S1 the 26 cerebelum regions extracted from the AAL template

| Index | Region | MNI |
| --- | --- | --- |
| 1 | Cerebelum_Crus1_L | [-36,-67,-29] |
| 2 | Cerebelum_Crus1_R | [37,-67,-29] |
| 3 | Cerebelum_Crus2_L | [-29,-73,-38] |
| 4 | Cerebelum_Crus2_R | [32,-69,-40] |
| 5 | Cerebelum_3_L | [-9,-37,-19] |
| 6 | Cerebelum_3_R | [12,-34,-19] |
| 7 | Cerebelum_4_5_L | [-15,-43,-17] |
| 8 | Cerebelum_4_5_R | [17,-43,-18] |
| 9 | Cerebelum_6_L | [-23,-59,-22] |
| 10 | Cerebelum_6_R | [24,-58,-23] |
| 11 | Cerebelum_7b_L | [-32,-60,-45] |
| 12 | Cerebelum_7b_R | [33,-63,-48] |
| 13 | Cerebelum_8_L | [-25,-55,-48] |
| 14 | Cerebelum_8_R | [25,-56,-49] |
| 15 | Cerebelum_9_L | [-11,-49,-46] |
| 16 | Cerebelum_9_R | [9,-49,-46] |
| 17 | Cerebelum_10_L | [-23,-34,-42] |
| 18 | Cerebelum_10_R | [26,-34,-41] |
| 19 | Vermis_1_2 | [0.76,-39,-20] |
| 20 | Vermis_3 | [1.38,-40,-11] |
| 21 | Vermis_4_5 | [1.22,-52,-6] |
| 22 | Vermis_6 | [1.14,-67,-15] |
| 23 | Vermis_7 | [1.14,-72,-25] |
| 24 | Vermis_8 | [1.15,-64,-34] |
| 25 | Vermis_9 | [0.86,-55,-35] |
| 26 | Vermis_10 | [0.36,-46,-32] |

Table S2 the 27 abnormal nodes extracted from the CONN

| Index | Region | MNI |
| --- | --- | --- |
| 1 | Cerebelum_3_R | [12,-34,-19] |
| 2 | Cerebelum_4_5_R | [17,-43,-18] |
| 3 | Cerebelum_6_R | [24,-58,-23] |
| 4  5  6 | Cerebelum_10_R  Cerebelum_Crus1_L  Inferior Frontal Gyrus Left | [26,-34,-41]  [-36,-66,-28]  [62,-35,32] |
| 7 | Lingual Gyrus Left | [-15,-67,-4] |
| 8 | Cingulum_Ant_L | [-5,35,13] |
| 9 | Cingulum_Ant_R | [7,37,15] |
| 10 | Precuneus_L | [-8,-56,48] |
| 11 | Precuneus_R | [8,-56,44] |
| 12 | Cingulum_Post_L | [-6,-43,24] |
| 13 | Cingulum_Post_R | [6,-42,22] |
| 14 | SupraMarginal_L | [-56,-33,30] |
| 15 | SupraMarginal_R | [56,-32,34] |
| 16 | Frontal_Sup_Medial_L | [-6,49,31] |
| 17 | Temporal_Pole_Mid_R | [43,14,-32] |
| 18 | Frontal_Sup_L | [-19,35,42] |
| 19 | Temporal_Mid_L | [-56,-34,-2] |
| 20 | Temporal_Mid _R | [56,-37,-1] |
| 21 | Angular_R | [45,-60,38] |
| 22 | Angular_L | [-45,-60,36] |
| 23 | Cingulum_Mid_L | [-6,-15,42] |
| 24 | Cingulum_Mid_R | [7,-9,40] |
| 25 | FrontoParietal.PPC (R) | [52,-52,45] |
| 26 | Language.pSTG (L) | [-57,-47,15] |
| 27 | Language.pSTG (R) | [59,-42,13] |
